# The synthesis of nitrogen/sulfur co-doped TiO_2_ nanocrystals with a high specific surface area and a high percentage of {001} facets and their enhanced visible-light photocatalytic performance

**DOI:** 10.1186/1556-276X-7-590

**Published:** 2012-10-24

**Authors:** Wenjing Shi, Weiyi Yang, Qi Li, Shian Gao, Panju Shang, Jian Ku Shang

**Affiliations:** 1 Materials Center for Water Purification, Shenyang National Laboratory for Materials Science, Institute of Metal Research, Chinese Academy of Sciences, Shenyang, 110016, People’s Republic of China; 2 Department of Materials Science and Engineering, University of Illinois at Urbana-Champaign, Urbana, IL, 61801, USA

**Keywords:** TiO_2_ nanocrystals, Highly active {001} facets, Nitrogen/sulfur co-doped, Visible-light photocatalytic performance

## Abstract

Nitrogen/sulfur co-doped anatase TiO_2_ nanocrystals with a high specific surface area and a high percentage of {001} facets were synthesized by a solvent-thermal process followed by the calcination with thiourea at an optimum heat treatment temperature. Under current experimental conditions, the optimum heat treatment temperature was found at 300°C, which successfully introduced nitrogen and sulfur dopants into the anatase lattice to replace a small portion of oxygen atoms while preserving the geometry of these anatase TiO_2_ nanocrystals to maintain a high percentage of {001} facets. These nitrogen/sulfur co-doped anatase TiO_2_ nanocrystals demonstrated a largely enhanced light absorption in the whole visible-light range and exhibited much higher photocatalytic performance than both P25 TiO_2_ nanoparticles and anatase TiO_2_ nanocrystals with a high percentage of {001} facets under visible-light illumination.

## Background

The discovery of the photoelectrochemical splitting of water on TiO_2_ electrodes by Fujishima and Honda in 1972 started the fast development on semiconductor-based photocatalysts [1]. Due to its high chemical stability, good photoactivity, relatively low cost, and nontoxicity, TiO_2_ is regarded as the leading candidate among various semiconductor-based photocatalysts, especially for industrial use [2]. In 2008, Lu and co-workers [3] successfully synthesized anatase TiO_2_ sheets with 47% exposed {001} facets using hydrofluoric acid as a capping agent, and the average size of the crystal was around 1 to 2 μm. From then on, anatase TiO_2_ single crystals with controlled facets attract a lot of research interests [3-14]. Both theoretical and experimental studies demonstrate that anatase TiO_2_ with exposed {001} facets is more active than anatase TiO_2_ with thermodynamically stable facets.

However, most reported anatase TiO_2_ single crystals with exposed {001} facets were in the micrometer size range [3-8], so their specific surface area values were quite small. For example, in the report by Yang et al. [4], the BET specific surface area of anatase TiO_2_ single crystals with 64% {001} facets was just 1.6 m^2^/g, a mere 3.4% of that of P25 TiO_2_ nanoparticles (approximately 47 m^2^/g). Although the formation efficiency of active hydroxyl radicals on these anatase TiO_2_ single crystals was found to be approximately 4.5 times as that of P25 TiO_2_ per unit surface area upon irradiation, the hydroxyl radical formation efficiency of these anatase TiO_2_ single crystals was only 15% of that of P25 TiO_2_ for the same material amount. In addition, micrometer-sized materials usually could not disperse well in water, and a small specific surface area also limits the efficient contact of photocatalysts with contaminants. Thus, micrometer-sized anatase TiO_2_ single crystals with exposed {001} facets are not the optimized choice for a good photocatalytic performance. Another intrinsic limitation of most reported anatase TiO_2_ with exposed {001} facets is that they need to be activated under the ultraviolet light (*λ* < 400 nm) illumination due to the relatively large bandgap (approximately 3.2 eV), which seriously limits their solar efficiency.

To make a better use of the solar illumination, one approach to extend the absorption band edge of TiO_2_ from the ultraviolet to the visible-light region was to introduce transition metal dopants into TiO_2_[15-21]. Recently, anionic nonmetal dopants, such as nitrogen [22-24], carbon [25,26], sulfur [27,28], or fluorine [29], had also been extensively explored for visible-light photocatalysis. A few reports had recently been made to incorporate anion dopants (N, S, and C) into anatase TiO_2_ with exposed {001} facets [30-32]. However, these anion-doped anatase TiO_2_ single crystals with exposed {001} facets were still in the micrometer size range, which may be attributed to the hydrothermal synthesis processes adopted between precursors of TiN, TiS_2_, and TiC with aqueous HF solution, respectively. It was reported that nanosized anatase TiO_2_ crystals with a high percentage of {001} facets could be obtained by the adoption of a solvent-thermal process to replace the hydrothermal process due to the smoother reaction and the potential directional effect of alcohols [12]. Thus, by the combination of both solvent-thermal process and anion doping, anion-doped anatase TiO_2_ nanocrystals with exposed {001} facets may be created, which could be more desirable for an enhanced photocatalytic performance under visible-light illumination.

In this work, the morphology control technique and the anion-doping technique were combined to further enhance the visible-light-activated photocatalytic performance of anatase TiO_2_ single crystals with exposed {001} facets. By the adoption of the solvent-thermal process, nanosized anatase TiO_2_ crystals with a high percentage of {001} facets were obtained which largely enhanced their specific surface areas. Interestingly, a moderate visible-light activity was found in these nanosized anatase TiO_2_ crystals with a high percentage of {001} facets. It had been demonstrated that anion co-doping may provide better visible-light absorption and photocatalytic performance than TiO_2_ or singly doped TiO_2_ with either dopant [23,33-35]. To further enhance their visible-light activity, a nitrogen/sulfur co-doping was introduced into this material system by a proper heat treatment with thiourea to replace a small portion of oxygen atoms in the anatase lattice while preserving the exposed {001} facet morphology. Thus, nitrogen/sulfur co-doped TiO_2_ nanocrystals with a high percentage of {001} facets and a large surface area were successfully created, which demonstrated largely enhanced visible-light absorbance and photocatalytic performance under visible-light illumination by the degradation of methylene blue (MB) and the disinfection of the bacteria *Escherichia coli* (*E. coli*), compared with commercial P25 TiO_2_ nanoparticles and pure anatase TiO_2_ nanocrystals with a high percentage of {001} facets.

## Methods

### Materials

Titanium(IV) fluoride (TiF_4_; 98%, Shanghai Darui Chemicals Co. Ltd., Shanghai, People' Republic of China) was used in this study as the precursor to provide both titanium and fluorine sources. *Tert*-butanol (C_4_H_10_O; ≥ 98%, Sinopharm Chemical Reagent Co., Ltd., Shanghai, People' Republic of China) was used as the solvent in the solvent-thermal process. Thiourea (CH_4_N_2_S; ≥ 99%, Sinopharm Chemical Reagent Co., Ltd.) was used to provide nitrogen and sulfur sources in the calcination process. Commercially available Degussa P25 TiO_2_ nanoparticles (Evonik Industries, Essen, Germany) were used for the comparison with nitrogen/sulfur co-doped TiO_2_ nanocrystals with highly active {001} facets on their visible-light photocatalytic performance.

### Synthesis

In a typical synthesis [12], 1.6 g of TiF_4_ was dissolved in 400 mL of *tert*-butanol under continuous stirring to obtain a transparent faint yellow solution. Then, the solution was transferred to the autoclave and allowed to alcoholize at 160°C for 3 days. The precipitated powders were filtered, washed with 0.1 M NaOH solution [10] to remove residual solvent for three times, and then dried at 50°C overnight to obtain the as-prepared TiO_2_ powders, which was denoted as T0. For the introduction of nitrogen/sulfur co-doping, the as-prepared TiO_2_ powders were mixed with thiourea [23,35] at a 2:1 weight ratio and then calcinated for 2 h at different temperatures of 300°C, 400°C, and 500°C, respectively, in a sealed tubular furnace. The obtained samples were denoted as T3, T4, and T5, correspondingly.

### Characterization

X-ray diffraction (XRD) experiments were conducted on a D/MAX-2004 X-ray powder diffractometer (Rigaku Corporation, Tokyo, Japan) with Ni-filtered Cu (*λ* =0.15418 nm) radiation at 56 kV and 182 mA to analyze the crystal structure and crystallite size of obtained powder samples. Their morphologies were examined by transmission electron microscopy (TEM) on a JEOL 2010 TEM (JEOL Ltd., Tokyo, Japan) operated at 200 kV, with a point-to-point resolution of 0.28 nm. TEM samples were prepared by dispersing a thin film of these powder samples on Cu grids. Their BET specific surface area values were measured by the N_2_ adsorption/desorption isotherm with an Autosorb-1 Series Surface Area and Pore Size Analyzer (Quantachrome Instruments, Boynton Beach, FL, USA). X-ray photoelectron spectroscopy (XPS) measurements were made using an ESCALAB250 X-ray photoelectron spectrometer (Thermo Fisher Scientific Inc., Waltham, MA, USA) with an Al K anode (1486.6 eV photon energy, 300 W). The UV–vis spectra of these powders were measured on a UV-2550 spectrophotometer (Shimadzu Corporation, Kyoto, Japan).

### Photocatalytic degradation of MB

MB (Acros Organics, Morris Plains, NJ, USA) was used as a model organic pollutant for the static photocatalytic degradation experiment under visible-light illumination. A powder sample was placed at the bottom of a 50 × 10-mm petri dish, and 4 ppm of MB solution was added into the petri dish at a fixed concentration of 1 mg photocatalyst/mL solution. Samples T0 and T3 were used in the photocatalytic degradation of the MB experiment, and P25 TiO_2_ powder was also used for comparison purposes under the same experimental conditions. The covered petri dishes were illuminated by a 300-W xenon lamp (PLS-SXE300, Beijing PerfectLight Technology Co., Ltd., Beijing, People's Republic of China), which has a glass filter to ensure a zero light intensity below 400 nm. The light intensity striking the MB solution was *ca.* 10 mW/cm^2^, as measured by a Multi-Sense optical radiometer (Beijing Normal University Photoelectricity Instruments Plant, Beijing, China). The visible-light illumination time varied from 5 to 30 min. After recovering the photocatalyst by centrifugation, the light absorption of the clear solution was measured by a UV-2550 spectrophotometer (Shimadzu Corporation).

### Photocatalytic inactivation of the bacteria *E. coli*

Wild-type *E. coli* AN 387 (ATCC 15597, the American Type Culture Collection, Manassas, VA, USA) were used for the photocatalytic inactivation experiment. After overnight culture, the cells were diluted to a cell suspension (*ca.*10^7^ cfu/mL) in buffer solution (0.05 M KH_2_PO_4_ and 0.05 M K_2_HPO_4_, pH 7.0) prior to the photocatalytic inactivation. All solid or liquid materials were autoclaved for 30 min at 121°C before use. For *E. coli* inactivation under visible-light illumination, the same xenon lamp was used, and the light intensity striking the cell suspensions was also at *ca.* 10 mW/cm^2^. In the photocatalytic inactivation experiment, the aliquot of the 10-mL *E. coli* cell suspension was pipetted into a sterile 50 × 10-mm petri dish with the photocatalytic powder sample placed in the bottom at a fixed concentration of 1 mg photocatalyst/mL solution. Samples T0 and T3 were used in the photocatalytic inactivation experiments, and the P25 TiO_2_ powder was also used for comparison purposes under the same experimental conditions. At regular time intervals, 100 μL of aliquots of the powder-treated cell suspensions was withdrawn in sequence. After appropriate dilutions in the buffer solution, aliquots of 100 μL were spread onto an agar medium plate and incubated at 37°C for 15 h. The number of viable cells in terms of colony-forming units was counted. Analyses were in duplicates, and control runs were carried out each time under the same experiment conditions but without any photocatalytic materials.

## Results and discussion

### Crystal structure and morphology of nitrogen/sulfur co-doped TiO_2_ nanocrystals

**Figure 1:**
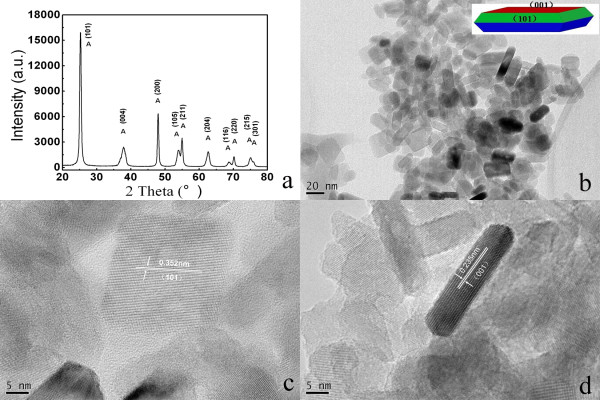
**Crystal structure and morphology of the as-prepared pure TiO**_**2**_**nanocrystals.** (**a**) XRD pattern, (**b**) TEM image, and (**c**, **d**) HRTEM images of as-prepared pure TiO_2_ nanocrystals with a high percentage of {001} facets (note that the insert in (b) demonstrated the schematic geometry of as-prepared pure TiO_2_ nanocrystals).

Figure 1 shows the crystal structure and morphology of the as-prepared pure TiO_2_ nanocrystals (T0). Figure 1a displays the XRD pattern of T0 synthesized by the solvent-thermal process, which clearly demonstrates that all XRD peaks belonged to anatase TiO_2_ (JCPDS No. 21–1272; space group: I41/amd(141)) with no rutile phase observed. Figure 1b shows the TEM observation of T0, which demonstrates that these nanocrystals were well faceted; square, hexagon, and rhombus shapes could be observed due to their different orientations. The high-resolution TEM (HRTEM) image of a single crystal with the rhombus shape (Figure 1c) demonstrates clear atomic planes with a lattice spacing of 0.352 nm, which correspond to the {101} lattice planes of anatase TiO_2_. Figure 1d shows the HRTEM image of the side view of a single crystal. Atomic planes with a lattice spacing of 0.235 nm could be easily observed, which correspond to the {001} lattice planes of anatase TiO_2_. From the HRTEM image observations, {001} lattice planes of the as-prepared pure TiO_2_ nanocrystals were parallel to their top and bottom planes. Thus, exposed {001} facets were present in these as-prepared pure TiO_2_ nanocrystals. From TEM/HRTEM observations and the symmetries of anatase TiO_2_ nanocrystals [3], the schematic geometry of the as-prepared pure TiO_2_ nanocrystals could be described as the insert of Figure 1b. Their side length was approximately 20 to 40 nm, and their thickness was approximately 6 to 10 nm. From their size and geometry, the percentage of exposed highly reactive {001} facets in these as-prepared pure TiO_2_ nanocrystals could be calculated by the method described by Zhu et al. [12]. The calculation was made on 20 TiO_2_ nanocrystals, and the average percentage of {001} facets was determined at approximately 60%.

**Figure 2:**
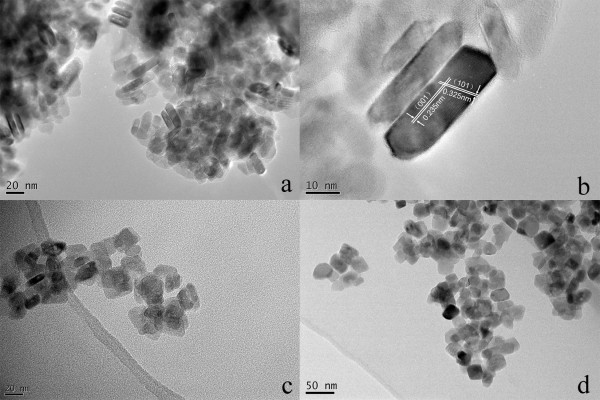
**Morphological evolution of T3, T4, and T5.** (**a**, **b**) TEM and HRTEM images of sample T3, respectively. (**c**, **d**) TEM images of samples T4 and T5, respectively.

To introduce nitrogen/sulfur co-doping, these as-prepared pure TiO_2_ nanocrystals were calcinated with thiourea at different temperatures of 300°C, 400°C, and 500°C, respectively, in a sealed tubular furnace. Figure 2 demonstrates the morphological evolution of T3, T4, and T5. After the heat treatment at 300°C, sample T3 kept the well-faceted geometry as the sample T0 (Figure 2a). {001} lattice planes of T3 were still parallel to their top and bottom planes, so the sample T3 also had exposed highly reactive {001} facets (Figure 2b). Compared with that of the sample T0, the side length of T3 decreased, while the thickness of T3 increased. With the increase of the calcination temperature to 400°C and 500°C, a clear geometry change could be observed for the samples T4 and T5 as demonstrated in Figure 2c,d, respectively. The well-faceted geometry of T0 gradually disappeared, and nanoparticles with non-uniform shapes appeared.

The XRD patterns of T3, T4, and T5 (not shown) demonstrate that all XRD peaks still belonged to anatase TiO_2_ (JCPDS No. 21–1272; space group: I41/amd(141)) with no rutile phase observed. Thus, the calcinations of T0 with thiourea from 300°C to 500°C did not change the crystal structure of the obtained TiO_2_ nanocrystals. However, the intensity of the (101) peak steadily increased with the increase of the calcination temperature, which suggests that the thermally stable {101} facets were enlarged. This observation is consistent with the TEM results. Thus, the calcination temperature should be carefully controlled to maintain the exposed highly reactive {001} facets. Under the current experimental conditions, the calcination temperature of 300°C is appropriate to maintain the exposed highly reactive {001} facets. BET specific surface area values of the samples T0 and T3 were measured. Because its size was in the nanometer range, the sample T0 had a large specific surface area of approximately 99 m^2^/g, which could largely enhance its contact efficiency with contaminants. After being calcinated with thiourea at 300°C, the specific surface area of T3 showed only a moderate decrease to approximately 70 m^2^/g. Thus, the sample of T3 also had a large specific surface area, which is desirable for its photocatalytic performance.

### Chemical composition of nitrogen/sulfur co-doped TiO_2_ nanocrystals

**Figure 3:**
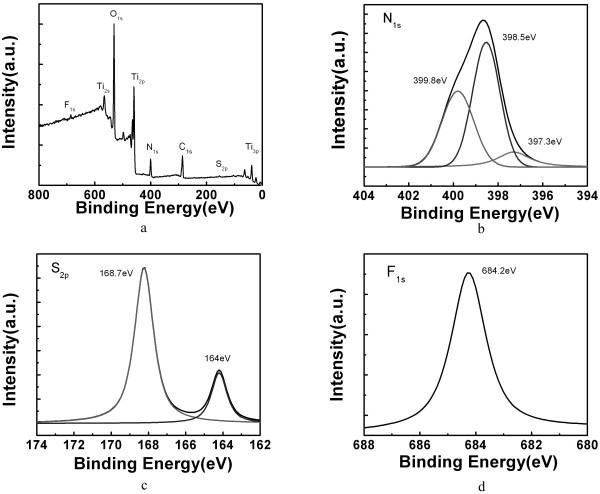
**XPS survey spectrum and high-resolution XPS scan spectra.** (**a**) XPS survey spectrum and high-resolution XPS scan spectra over (**b**) N 1*s*, (**c**) S 2*p*, and (**d**) F 1*s* peaks of the sample T3.

To investigate the chemical composition of TiO_2_ nanocrystals with a high percentage of {001} facets after calcinations with thiourea, XPS investigations were conducted to obtain semi-quantitative composition data. Figure 3a shows a representative XPS survey spectrum of T3 (calcinations at 300°C), which clearly demonstrated the existence of Ti, O, N, S, F, and C. The existence of the C 1*s* peak could be attributed to the widespread presence of carbon in the environment. The relative element composition ratio was determined by multiplex high-resolution scans over Ti 2*p*, O 1*s*, N 1*s*, S 2*p*, and F 1*s* spectral regions. Multiplex high-resolution scans over N 1*s*, S 2*p*, and F 1*s* spectral regions are shown in Figure 3b,c,d, respectively. Figure 3b demonstrates that the N 1*s* peak could be best fitted by a combination of three N 1*s* peaks at 397.3, 398.5, and 399.8 eV, respectively. The N 1*s* peak at 397.3 eV is associated with Ti-N bonding [22-24], which clearly demonstrated that nitrogen atoms were doped into the anatase lattice and replaced a small portion of oxygen atoms during the synthesis process, while the N 1*s* peaks at 398.5 and 399.8 eV represent some organic impurities or chemisorb N_2_ on the sample surface. The doped N accounted for about 10.5% of the total N; thus, the doped N/Ti atomic ratio was determined at approximately 8.9%. This observation is different from some previous reports on nitrogen-doped TiO_2_ with a high percentage of {001} facets wherein no doped N was found to form the Ti-N bonding [30,36], which may be attributed to the different doping methods/conditions used. Figure 3c demonstrates that the S 2*p* peak could be best fitted by a combination of two S 2*p* peaks at 164.0 and 168.7 eV, respectively. The S 2*p* peak at 164.0 eV corresponded to the Ti-S bonding, while the S 2*p* peak at 168.7 eV corresponded to the surface S atoms adsorbed as SO_2_ molecules [27,28]. The doped S accounted for about 34.8% of the total S; thus, the doped S/Ti atomic ratio was determined at approximately 1.0%. Figure 3d demonstrated that the F 1*s* peak position was at 684.2 eV, which corresponded to F^−^ ions physically adsorbed on the sample surface [3,33,34]. From the above XPS analysis results, it is clear that nitrogen/sulfur co-doping was introduced after calcinations with thiourea and that nitrogen/sulfur co-doped TiO_2_ nanocrystals with a high percentage of {001} facets were successfully created by our approach. XPS analysis was also conducted on T4 and T5 samples with a higher calcination temperature, which showed a similar result of nitrogen/sulfur co-doping. For the sulfur dopant, the doping concentration did not change much. However, an obvious loss of nitrogen dopant was observed with the increase of the calcination temperature. The doped N/Ti atomic ratio sharply dropped from 8.9% for T3 to 0.67% for T5, which could largely deteriorate its visible-light absorption capability.

### Optical properties of nitrogen/sulfur co-doped TiO_2_ nanocrystals

**Figure 4:**
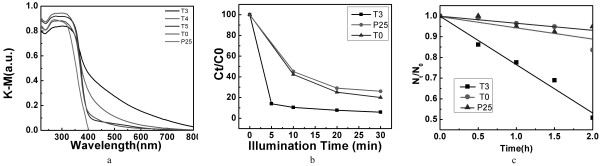
**Optical absorbance spectra, residue MB percentage, and survival ratio.** (**a**) UV-visible light absorbance spectra of T0, T3, T4, and T5, compared with that of P25 TiO_2_ nanoparticles. (**b**) The residue MB percentage vs. treatment time in treated MB solutions by P25 TiO_2_ nanoparticles, the sample T0, and the sample T3 under visible-light illumination, respectively. (**c**) Survival ratio of *E. coli* cells vs. visible-light (*λ* > 400 nm) illumination time with T3, T0, P25, respectively.

The optical properties of nitrogen/sulfur co-doped TiO_2_ nanocrystals with a high percentage of {001} facets were investigated by measuring their diffuse reflectance spectra. The optical absorbance was then approximated from the reflectance data by the Kubelka-Munk function, as given by Equation 1:

(1) $$F\left(R\right)=\frac{{\left(1-R\right)}^{2}}{2R}\text{,}$$

where *R* is the diffuse reflectance [37]. Figure 4a shows the light absorbance curves (in terms of Kubelka-Munk equivalent absorbance units) of nitrogen/sulfur co-doped TiO_2_ nanocrystals with {001} facets (T3, T4, and T5), which were compared with that of pure TiO_2_ nanocrystals without doping (T0) and commercially available P25 TiO_2_ nanoparticles. P25 TiO_2_ nanoparticles are widely used in the photocatalyst studies as a model TiO_2_ with high photocatalytic performance. As expected, the light absorbance of P25 TiO_2_ had the characteristic spectrum with the fundamental absorbance stopping edge at approximately 400 nm, so its light absorbance was limited mainly in the UV light range. Pure TiO_2_ nanocrystals with a high percentage of {001} facets, however, demonstrated a moderate light adsorption capability from 400 to 500 nm, which had not been reported before. This observation may be attributed to the nanosize of the sample T0, which largely increased its {001} surface/bulk ratio. Ariga et al. [38] demonstrated using their scanning tunneling microscopy study that a surface state-mediated visible-light activity could occur on nanostructured TiO_2_ {001} facets. Thus, the largely enhanced {001} surface/bulk ratio of the sample T0, compared with that of micro-sized TiO_2_ single crystals with a high percentage of {001} facets, may lead to this observed visible-light absorbance. With nitrogen/sulfur co-doping, the visible-light absorbance of TiO_2_ nanocrystals with a high percentage of {001} facets was largely enhanced. T3 demonstrated a much higher light absorbance from approximately 380 to 800 nm than T0, which could be attributed to the anion-doping effect from the co-doping of nitrogen and sulfur. Thus, it should possess a largely enhanced photocatalytic performance than pure TiO_2_ nanocrystals with a high percentage of {001} facets. With the increase of the calcination temperature, the visible-light absorbance of T4 and T5 decreased gradually. T4 still possessed a good visible-light absorbance, while the light absorbance of T5 was similar with that of T0. This observation suggests that the calcination temperature for anion doping in our synthesis process is critical to the light absorbance of these samples. With the increase of the calcination temperature (over 300°C), the decomposition of the anion-doping source of thiourea accelerated, and more and more nitrogen and sulfur got lost without being doped into the anatase lattice. This observation is in agreement with the XPS measurement results that the anion-doping concentration decreased with the increase of the calcination temperature.

### Enhanced photodegradation of methylene blue and photocatalytic inactivation of *E. coli* by nitrogen/sulfur co-doped TiO_2_ nanocrystals under visible-light illumination

From the above studies of morphology, anion-doping concentration, and light absorption performance, it is clear that the sample T3 had the highest percentage of {001} facets, anion-doping concentration, and visible-light absorption. Thus, the visible-light photocatalytic activities of the nitrogen-doped TiO_2_ nanocrystal photocatalyst with a high percentage of {001} facets were evaluated with the sample T3 by its photocatalytic degradation of MB and photocatalytic inactivation of *E. coli* under visible-light illumination (*λ* > 400 nm) and compared with that of T0 and commercially available P25 TiO_2_ nanoparticle photocatalyst. Photocatalytic degradation of MB was conducted by exposing the MB solution with various photocatalysts under visible light for varying time intervals (from 5 to 30 min). After the centrifugation to recover photocatalysts, the light absorption of the clear solution was measured, and the remaining percentage of MB in the solution was calculated by the ratio between the light absorptions of photocatalyst-treated and photocatalyst-untreated MB solutions. Figure 4b shows the MB residue ratio changes in treated MB solutions with different treatment times. P25 TiO_2_ demonstrated a fair degradation performance on MB under visible-light illumination, which could be attributed to its mixed nature of both anatase and rutile phases of TiO_2_. Its superior dispersity in an aqueous environment also contributes to its efficient contact with organic pollutants. After a 30-min treatment, its degradation effect slowed down, and the residual MB percentage was approximately 30%. Without nitrogen doping, the sample T0 showed a slightly better degradation effect on MB compared with the P25 TiO_2_ nanoparticles under visible-light illumination, which could be attributed to its moderate light adsorption capability in the wavelength range from 400 to 500 nm as demonstrated in Figure 4a. The sample T3, however, demonstrated a much faster degradation effect on MB under visible-light illumination than both P25 TiO_2_ and T0. Within just 5 min, most of the MB in the solution was degraded, and the residual MB percentage dropped to just approximately 14%. After a 30-min treatment, the residual MB percentage was near zero, suggesting a complete degradation of MB.

The photocatalytic activity of sample T3 was further demonstrated by its bactericidal effect on the viability of *E. coli* cells and compared with that of sample T0 and P25 TiO_2_. The photocatalytic inactivation experiments on *E. coli* were conducted by exposing the cells suspended in the buffer solution with various photocatalysts under visible-light illumination (*λ* > 400 nm) for varying time intervals. The survival ratio of *E. coli* was determined by the ratio of *N*_t_/*N*_0_, where *N*_0_ and *N*_t_ are the numbers of colony-forming units at the initial time and each following time interval, respectively. Figure 4c shows the *E. coli* survival ratio under various treatments. P25 TiO_2_ showed no obvious bactericidal effect under visible-light illumination. After a 2-h treatment, the *E. coli* survival ratio was still around 95%. The sample T0 showed a slightly better photocatalytic inactivation effect on *E. coli* under visible-light illumination compared with P25 TiO_2_ nanoparticles. After a 2-h treatment, the *E. coli* survival ratio was around 85%. The sample T3 demonstrated a much better photocatalytic inactivation effect on *E. coli* under visible-light illumination. The survival ratio of *E. coli* showed a continuous decreasing trend with the increase of visible-light illumination time. After a 2-h treatment, the survival ratio of *E. coli* dropped to around 50%. This observation is in agreement with the photocatalytic degradation experiment results demonstrated in Figure 4b. Thus, the combination of the large specific area due to their nanosize, high percentage of active {001} facets, and enhanced visible-light adsorption due to nitrogen/sulfur co-doping optimized the photocatalytic performance of TiO_2_ under visible-light illumination.

## Conclusions

In summary, nitrogen/sulfur co-doped anatase TiO_2_ nanocrystals with a high specific surface area and a high percentage of {001} facets were successfully synthesized by a solvent-thermal process followed by the calcination with thiourea at an optimized heat treatment temperature. The solvent-thermal process reduced the crystal size of TiO_2_ single crystals with a high percentage of {001} facets to nanosize, which largely increased their specific surface area and the contact efficiency with contaminants compared with most previous reports. Both nitrogen and sulfur dopants were introduced into the anatase TiO_2_ lattice to replace a small portion of oxygen by calcinating the as-prepared TiO_2_ nanocrystals with thiourea at a proper temperature, which largely enhanced their light absorption in the visible-light range. Thus, with the combination of the large specific area due to their nanosize, high percentage of active {001} facets, and enhanced visible-light adsorption due to nitrogen/sulfur co-doping, the photocatalytic performance of TiO_2_ under visible-light illumination was optimized. As demonstrated by the photocatalytic degradation of MB and the photocatalytic inactivation of *E. coli* bacteria, nitrogen/sulfur co-doped TiO_2_ nanocrystals with a high specific surface area and a high percentage of {001} facets exhibited a much higher photocatalytic performance than P25 TiO_2_ nanoparticles and pure anatase TiO_2_ nanocrystals with a high percentage of {001} facets under visible-light illumination.

## Competing interests

The authors declare that they have no competing interests.

## Authors’ contributions

WS and WY carried out the synthesis, characterization, and photocatalytic degradation experiments and participated in the preparation of the manuscript. QL conceived of the study, participated in its design and coordination, and wrote the manuscript. SG and PS participated in the synthesis and characterization experiments. JKS participated in the design of the study and the preparation of the manuscript. All authors read and approved the final manuscript.
